# Intimate disclosure in online‐only friendships predicts adolescents' depressive symptoms

**DOI:** 10.1111/jora.70144

**Published:** 2026-01-28

**Authors:** Zelal Kilic, Anne J. Maheux, Jacqueline Nesi, Jennifer S. Silk, Sophia Choukas‐Bradley

**Affiliations:** ^1^ Department of Psychology University of Pittsburgh Pittsburgh Pennsylvania USA; ^2^ Department of Psychology and Neuroscience University of North Carolina at Chapel Hill Chapel Hill North Carolina USA; ^3^ Department of Psychiatry and Human Behavior Brown University Providence Rhode Island USA; ^4^ Bradley Hasbro Research Center Rhode Island Hospital Providence Rhode Island USA; ^5^ Department of Psychiatry University of Pittsburgh Pittsburgh Pennsylvania USA

**Keywords:** adolescence, depression, intimate disclosure, online‐only friendships

## Abstract

Adolescents frequently use social media to form online‐only friendships (OOFs; i.e., friendships that are exclusive to digital platforms), raising questions about their emotional impact. While OOFs may provide social support and increase well‐being, they could also displace in‐person relationships or increase exposure to online risks. This study examined longitudinal associations between OOFs, intimate disclosure within these friendships, and depressive symptoms among adolescents. A total of 1089 teens aged 13–19 (*M* = 15.65, *SD* = 1.19; 581 girls, 492 boys, and 16 another gender identity) completed online surveys as part of a longitudinal study investigating adolescent development at two timepoints: October 2019 (T1) and February 2020 (T2). Teens reported whether they had any OOFs, and, if so, levels of intimate disclosure within their OOFs, as well as depressive symptoms. There was a significant correlation between OOF status and depressive symptoms at T1 (*r* = .13, *p* < .01). However, T1 OOF status was not significantly associated with depressive symptoms at T2 (unstandardized *b* = 0.02*, p* = .64, *IRR* = 1.02), or vice versa (*OR =* 1.02*, p =* .055, *CI* = 1.00–1.05), controlling for age, gender, and baseline levels of the outcome variable. Higher T1 intimate disclosure with OOFs predicted higher T2 depressive symptoms (unstandardized *b =* 0.088, *p* = .011, *IRR* = 1.09), while T1 depressive symptoms did not significantly predict T2 intimate disclosure (unstandardized *b* = 0.012, *p* = .069, *CI* = −0.001–0.03), controlling for age, gender and baseline levels of the outcome variable. These results suggest that while the sole presence of OOFs is associated with depressive symptoms, intimate disclosure within these friendships may predict increases in depressive symptoms.

## INTRODUCTION

The prevalence of depressive symptoms among youth has increased exponentially over the last two decades, coinciding with the widespread and heavy use of digital media (Daly, 2022; Duffy et al., 2019; Orben, 2020; Twenge et al., 2018). Adolescent social media use has been described as a “double‐edged sword,” providing opportunities for both positive and negative mental health consequences (Choukas‐Bradley et al., 2023). Digital environments have transformed how adolescents form and maintain peer relationships. During adolescence, when peer relationships gain utmost importance, increased levels of social connectedness with friends can play a vital role in mitigating various mental health problems, particularly depression (Jose et al., 2012; Jose & Lim, 2014). With the advent of new digital platforms altering the dynamics of peer relationships, it is important to investigate both the risks and benefits of digital environments for youth depression. Researchers have recently noted that positive online peer experiences could offer protective effects against depressive symptoms, via providing social support, reducing feelings of isolation, and bolstering a sense of belonging (Angelini & Gini, 2024; Nesi, 2020; Berger et al., 2022; Ivie et al., 2020; Popat & Tarrant, 2023; Sala et al., 2024). One potential way in which social media may provide these buffering effects is through online‐only friendships (OOFs)—friendships that are exclusive to digital platforms. Yet, little is known about how OOFs may be associated with depressive symptoms over time. This study explores this question in a large sample of adolescents.

The transition from childhood to adolescence marks a period of increased risk for developing depression, with girls experiencing a more pronounced increase than boys (Daly, 2022; Hankin et al., 2015; Salk et al., 2017). Concomitantly, during the transition to adolescence, teens increasingly prioritize their peer relationships and experience heightened needs to belong in social groups (Bagwell & Bukowski, 2018). Indeed, interpersonal models of depression propose that negative peer experiences, such as peer rejection, lack of social support, or peer victimization are primary causes of depression (Coyne, 1976; Van Orden et al., 2010). Conversely, higher levels of perceived social connectedness are associated with positive outcomes, including better mental health (Neel & Fuligni, 2013; Oberle et al., 2011). Therefore, it is crucial to understand the ways in which online friendships might be associated with depressive symptoms during this sensitive period of development.

Despite the growing interest in the links between digital media and adolescent depression (Cunningham et al., 2021; Hilty et al., 2023; Ivie et al., 2020), little is known about how the use of social media may be related to the development of depression over time, largely due to methodological limitations (Orben & Przybylski, 2019). For instance, most studies have relied on cross‐sectional designs and many have used a global measure of screen time, which cannot capture the nuanced ways teens use social media to connect with peers and seek support, such as by forming OOFs with other youth who are not known offline (Coyne et al., 2021; McAllister et al., 2021; Twenge et al., 2018; Valkenburg et al., 2022). It is possible that OOFs provide additional layers of social support for adolescents, particularly those who are at higher risk for depression (Massing‐Schaffer et al., 2022). For example, perhaps due to the increased accessibility and availability of communication online—as proposed by the transformation framework (Nesi et al., 2018a, 2018b)—these interactions could especially benefit teens who experience difficulties in their daily in‐person interpersonal interactions, providing an avenue for more positive and meaningful social interactions that are easier to maintain (Ybarra et al., 2015).

Although studies specific to OOFs are limited, a systematic review by Marchant et al. (2017) showed that online forums can provide avenues for youth to feel connected with others and seek social support, suggesting that primarily digital interactions may have many beneficial qualities. Adolescents who struggle with depressive symptoms may turn to these friendships for engagement and social support that they lack otherwise. One longitudinal study spanning over a year by Massing‐Schaffer et al. (2022) found that teens who experienced higher levels of offline friendship stress showed decreases in their suicidal ideation if they had at least one OOF, compared with their counterparts who did not report having OOFs.

While these initial findings point to the potential protective roles of OOFs, such friendships may also pose the risk of supplanting teens' in‐person interactions, potentially limiting their opportunities to build social skills or long‐term friendships, and perhaps indirectly contributing to higher levels of depressive symptoms. One way in which OOFs may be linked differently to adolescent well‐being may be through the amounts of intimate disclosure that take place within these friendships. As opposed to the interpersonal interactions common in public contexts, such as online forums, OOFs may be characterized by intimate disclosure—an interpersonal process in which teens share personal and intimate information about themselves (Omarzu, 2000).

However, a systematic review on self‐disclosure found that while overall self‐disclosure seems to be beneficial for well‐being, online self‐disclosure appears to be less fulfilling than in‐person self‐disclosure (Towner et al., 2022). Importantly, the authors also highlight that anxious youth specifically engage in more self‐disclosure online. This could suggest that teens who struggle with mental health or interpersonal relationships in‐person might prefer digital platforms—perhaps due to increased cue absence, anonymity, and availability of digital communication—but the direction of these associations seems to be unknown. Therefore, while investigating potential links between OOFs and depression, it is also important to investigate whether intimate disclosure with online‐only friends may be linked to future depressive symptoms and vice versa.

In the current paper, we provide initial insights into longitudinal associations between OOFs and depressive symptoms over the course of 4 months, to examine one nuanced way that adolescents engage in social media and its prospective links with depressive symptoms. Furthermore, among adolescents who report having OOFs, we investigate whether there are associations between intimate disclosure and depressive symptoms. While the only prior study investigating OOFs and depressive symptoms spanned over 1 year, it did not investigate this relationship longitudinally (Massing‐Schaffer et al., 2022). Given that OOFs represent a new and potentially important interpersonal context for adolescent development and mental health, we argue that it is important to continue to investigate OOFs in different samples and across different time scales. Substantial prior research reveals that depressive symptoms among youth can fluctuate over periods shorter than 1 year, including lags of about 4 months, similar to the current study (Burnell et al., 2025; Gentzler et al., 2023; Kirchner et al., 2022; Li et al., 2023). Moreover, a recent *Psychological Bulletin* paper discussed the need for shorter‐term longitudinal studies to better detect friendship influence processes (Giletta et al., 2021). The hypotheses are as follows:

1. Because OOFs can act as a source of social support, we hypothesized that teens who report having OOFs at Time 1 (T1) will report lower levels of Time 2 (T2) depressive symptoms compared with youth who report no T1 OOFs.
2. Because teens with higher levels of depressive symptoms may turn to OOFs to build social connections, we hypothesized that teens with higher levels of T1 depressive symptoms will be more likely to report a T2 OOF, relative to those with lower levels of depressive symptoms at T1.
3. For those who report having OOFs at T1, higher levels of T1 intimate disclosure within these OOFs will predict lower levels of T2 depressive symptoms.
4. For those who report having OOFs at T1, higher levels of T1 depressive symptoms will predict higher levels of T2 intimate disclosure.

## METHOD

### Participants and procedure

At baseline, a total of 1587 adolescents aged 13–19 (*M*_*age*_ = 15.62, *SD*_*age*_ = 1.21; 810 girls, 756 boys, and 21 another gender identity) from a large school district in Florida were recruited through Character Lab Research Network, a consortium of schools across the U.S. working in collaboration with researchers. We excluded participants missing data on key variables, specifically participants who did not respond to the questions about depressive symptoms (*n* = 144) or OOFs (*n* = 451); this resulted in our final analytic sample consisting of a total of 1089 teens aged 13–19 (*M*_*age*_ = 15.65, *SD*_*age*_ = 1.19; 581 girls, 492 boys, and 16 another gender identity). Participants completed online surveys at two time points (October 2019 and February 2020; T1 and T2, respectively) via Qualtrics during regular school hours, as part of a longitudinal study of adolescent development. The 4‐month lag between assessments was determined by school schedule; a third time point was originally scheduled for Spring 2020 but was canceled due to the onset of the COVID‐19 pandemic and consequent school closures. Data from school information systems included participants' age, eligibility for free or reduced‐price lunch (to be used as a proxy for socioeconomic status), and race/ethnicity (as assessed via a “select all that apply” measure). The teens in the final analytic sample identified as follows: 79.4% White, 9.4% Black, 6.6% Asian, and 4.6% another racial/ethnic identity or multiracial; 54.4% of the sample also identified as Hispanic/Latinx ethnicity and 33.2% were eligible for free or reduced‐price lunch.

### Measures

#### Depressive symptoms

Participants completed the Short Mood and Feelings Questionnaire (SMFQ; Sharp et al., 2006) as a measure of depression at both time points. SMFQ is a 13‐item self‐report questionnaire assessing depressive symptoms over the past 2 weeks, with items, such as “I felt miserable or unhappy” and “I didn't enjoy anything at all” rated on a scale of 0 (not true), 1 (sometimes true), and 2 (mostly true). Total scores range from 0 to 26, with higher scores indicating higher levels of depressive symptoms. The internal reliability for SMFQ was excellent in the current sample (Cronbach alphas were .93 at T1 and .95 at T2).

#### OOF status and intimate disclosure

Teens reported whether they had any OOFs by answering the following question: “Do you have any online friends who you have not met in person?” with response options “Yes” and “No.” Teens who reported having OOFs were asked five follow‐up questions about their intimate disclosure within these relationships, partially adapted from the Intimate Disclosure subscale of the Network of Relationship Inventory, and previously used to assess intimate disclosure in OOFs (Furman & Buhrmester, 1985; Massing‐Schaffer et al., 2022): “How much do you talk about everything with these online friends?”, “How much do you share your secrets and private feelings with these online friends?”, “How much do you talk to these online friends about things you don't want others to know?”, and “How much do you talk to these online friends about issues related to mental health?” and were rated on a scale of 1 (none) to 5 (extremely much). These items reflect the breadth and depth dimensions of intimate disclosure originally proposed by Omarzu (2000). Additionally, participants were asked, “How close are you to these online friends, compared to your in‐person friends (in‐person friends are people who you know offline, even if you also communicate with them online)?”, which was rated on a scale of 1 (much closer to online friends) to 5 (much closer to in‐person friends). The last item was reverse‐scored, and all five items were used to create a mean score as an index of intimate disclosure, with higher scores indicating higher levels of intimate disclosure. Cronbach's alphas for intimate disclosure were .84 at T1 and .85 at T2, showing good internal reliability.

#### Covariates

Age and gender identity were used as covariates in all models given the developmental trajectories of depressive symptoms based on age and gender (Angold et al., 1998; Salk et al., 2017). Participants were asked to self‐report their gender identity on the traditional gender binary as female or male, with the option for “other” or “prefer not to say” as well. However, due to the low frequency of participants (16 individuals, 1.5%) identifying outside of the gender binary in the current sample, results from this category will be interpreted cautiously.

### Analytic approach

All data preparation and statistical analyses were conducted using R Version 4.4.1 (R, 2024). To account for a nonnormal and zero‐inflated distribution of depressive symptoms at T2, zero‐inflated negative binomial models were used to test Hypotheses 1 and 3, logistic regression for the binary OOF variable in Hypothesis 2, and linear regression for Hypothesis 4. Specifically, to test Hypothesis 1, we used a zero‐inflated negative binomial model to examine whether OOF status at T1 predicted depressive symptoms at T2 in the full sample; more specifically, this two‐part model examined whether reporting one or more OOFs at T1 predicted the presence of any depressive symptoms at T2 (i.e., zero‐inflated model), and among those with any T2 depressive symptoms, whether T1 OOF status predicted the number of depressive symptoms (i.e., count model). For Hypothesis 2, we tested whether depressive symptoms at T1 predicted OOF status at T2 in a logistic regression model using the full sample. Hypotheses 3 and 4 focused on intimate disclosure within OOFs, among the subsample of participants who reported any OOFs at T1. To test Hypothesis 3, we used a zero‐inflated negative binomial model to examine whether intimate disclosure at T1 predicted depressive symptoms at T2 (as in Hypothesis 1, we modeled both the presence of any depressive symptoms as well as their count). Finally, for Hypothesis 4, we used a linear regression model to examine whether depressive symptoms at T1 predicted intimate disclosure within OOFs at T2. All analyses included age, gender, and T1 levels of the outcome variables as covariates.

## RESULTS

Descriptive statistics and bivariate correlations for all study variables are shown in Table 1. Girls reported significantly higher levels of depressive symptoms than boys at both time points, and boys were significantly more likely to report having OOFs than girls at T1. Cross‐sectionally, having OOFs was positively correlated with depressive symptoms at T1 for the full sample (*r* = .13, *p* < .01).

**TABLE 1:** Means, standard deviations, gender comparisons, and correlations with confidence intervals.

Variables	Girls	Boys	*t, p*	1	2	3	4	5	6
	*n* = 581	*n* = 492							
	*M* (*SD*)	*M* (*SD*)							
1. Age	15.6 (1.22)	15.7 (1.20)	−1.79, .073						
2. Depressive symptoms at T1	9.76 (7.36)	6.60 (6.60)	7.42, <.001***	.01					
				[−.05, .07]					
3. OOF status at T1	0.49 (0.5)	0.63 (0.48)	−4.66, <.001***	.03	.13***				
				[−.03, .09]	[.07, .19]				
4. Intimate disclosure at T1	2.71 (0.95)	2.71 (0.91)	0.31, .76	.12**	.24***	^a^			
				[.04, .20]	[.16, .31]				
5. Depressive symptoms at T2	9.72 (7.62)	6.89 (7.55)	5.95, <.001***	.01	.62***	.07	.24***		
				[−.07, .08]	[.58, .66]	[−.00, .14]	[.14, .33]		
6. OOF status at T2	0.50 (0.5)	0.53 (0.5)	−0.79, .43	.02	.15***	.47***	.20***	.06	
				[−.05, .10]	[.07, .22]	[.41, .52]	[.10, .29]	[−.01, .14]	
7. Intimate disclosure at T2	2.93 (0.90)	2.95 (0.91)	−0.07, .94	.13*	.20***	.04	.57***	.26***	^a^
				[.02, .23]	[.10, .31]	[−.07, .15]	[.48, .65]	[.15, .35]	

*Note*: *M* and *SD* are used to represent mean and standard deviation, respectively. Means, standard deviations, and comparisons across gender only include adolescents who identified with a binary gender identity; correlations include the full sample. Values in square brackets indicate the 95% confidence interval for each correlation. The confidence interval is a plausible range of population correlations that could have caused the sample correlation (Cumming, 2014). T1 = Time 1 (Baseline), T2 = Time 2, OOF Status = online‐only friendship status (no = 0, yes = 1).

^a^ Within‐time‐point bivariate correlations between OOF Status and Intimate Disclosure are not possible to compute (i.e., OOF Status at T1 and Intimate Disclosure at T1; OOF Status at T2 and Intimate Disclosure at T2) because participants reported Intimate Disclosure scores only if they reported OOFs (i.e., if a participant did not report having an online‐only friend, then their intimate disclosure score was missing, and if they reported having an online‐only friend, their OOF Status necessarily = 1).

* *p* < .05.

** *p* < .01.

*** *p* < .001.

### Hypotheses 1 and 2: OOF status and depressive symptoms

Having OOFs at T1 was not significantly associated with levels of depressive symptoms at T2 in the count model (unstandardized *b* = 0.02, *p* = .64, *IRR* = 1.02) or the zero‐inflated model (unstandardized *b* = 0.37, *p* = .083, *OR* = 1.45). T1 depressive symptoms were a marginally significant predictor of T2 OOF status (*OR* = 1.04, *p =* .055, *CI* = 1–1.05), after accounting for age, gender, and T1 OOF status. See Tables 2 and 3 for full model results.

**TABLE 2:** Negative binomial model predicting depressive symptoms at Time 2 from OOF status at Time 1.

Predictors	*B*	*SE*	*p*
*Count model*			
(Intercept)	1.69	0.34	<.001***
Depressive symptoms at T1	0.05	0.00	<.001***
OOF status at T1	0.02	0.05	.643
Gender: Male	−0.05	0.06	.363
Gender: Another gender	0.36	0.17	.037*
Age	0.01	0.02	.777
*Zero‐inflated model*			
(Intercept)	−1.98	1.41	.162
Depressive symptoms at T1	−0.19	0.03	<.001***
OOF status at T1	0.37	0.21	.083
Gender: Male	0.55	0.22	.012*
Gender: Another gender	−14.57	1055.7	.989
Age	0.074	0.10	.413
Observations	774		

Abbreviations: OOF, online‐only friendship; T1 = Time 1 (Baseline).

*** *p* < .001;

* *p* < .05.

**TABLE 3:** Logistic regression model predicting online‐only friendship status at Time 2 from depressive symptoms at Time 1.

Predictors	Odds ratios	*SE*	*CI*	*p*
(Intercept)	0.23	1.15		.204
Depressive symptoms at T1	1.04	0.01	1, 1.05	.055
OOF status at T1	7.7	0.18	5.22, 10.49	<.001***
Gender: Male	1.09	0.18	0.66, 1.36	.773
Gender: Another gender	3.17	0.74	0.68, 12.46	.148
Age	1.04	0.07	0.88, 1.17	.852
Observations	694			
Log likelihood	−398.65			
AIC value	809.29			

Abbreviations: AIC, Akaike information criterion; OOF, online‐only friendship; T1, Time 1 (Baseline).

*** *p* < .001.

### Hypotheses 3 and 4: Intimate disclosure and depressive symptoms

Intimate disclosure was a significant predictor of depressive symptoms at T2, but in the opposite direction as hypothesized in Hypothesis 3 (unstandardized *b =* 0.08, *p* = .02, *IRR* = 1.09 in the count model; unstandardized *b =* −0.10, *p* = .52, *OR* = 0.90 in the zero‐inflated model; see Table 4 for full model results). This means that a one‐unit increase in intimate disclosure was associated with a 9.2% increase in the expected count of depressive symptoms at follow‐up. Higher levels of depressive symptoms at T1 were not a significant predictor of intimate disclosure within OOFs at T2 (unstandardized *b* = 0.012, *p* = .069, *CI* = −0.001–0.03; see Table 5 for full model results).

**TABLE 4:** Negative binomial model predicting depressive symptoms at Time 2 from OOF intimate disclosure at Time 1.

Predictors	*B*	*SE*	*p*
*Count model*			
(Intercept)	2.00	0.42	<.001***
Depressive symptoms at T1	0.05	0.00	<.001***
OOF intimate disclosure at T1	0.08	0.04	.015*
Gender: Male	−0.04	0.07	.52
Gender: Another gender	0.47	0.18	.01*
Age	−0.02	0.03	.46
*Zero‐inflated model*			
(Intercept)	−2.00	1.91	.30
Depressive symptoms at T1	−0.16	0.03	<.001***
OOF intimate disclosure at T1	−0.10	0.15	.52
Gender: Male	0.81	0.30	.006**
Gender: Another gender	−14.40	1224.64	.99
Age	0.10	0.12	.44
Observations	405		

Abbreviations: OOF, online‐only friendship; T1, Time 1 (Baseline).

*** *p* < .001;

** *p* < .01;

* *p* < .05.

**TABLE 5:** Linear regression model predicting OOF intimate disclosure at Time 2 from depressive symptoms at Time 1.

Predictors	*B*	*SE*	*t*	*p*
(Intercept)	0.92	0.66	1.39	.17
Depressive symptoms at T1	0.01	0.01	0.72	.48
OOF intimate disclosure at T1	0.54	0.05	10.34	<.001***
Gender: Male	0.04	0.10	0.38	.71
Gender: Another gender	0.19	0.33	0.57	.57
Age	0.02	0.04	0.55	.58
Observations	249			
*R* ^2^/*R* ^2^ adjusted	0.331/0.318			

Abbreviations: OOF, online‐only friendship; T1, Time 1 (Baseline).

*** *p* < .001.

## DISCUSSION

The current study investigated adolescents' reports of OOFs, the intimate disclosure within those friendships, and the potential longitudinal links with adolescents' depressive symptoms 4 months later, in a large sample of adolescents. Our findings indicated that the presence of OOFs did not predict lower levels of depressive symptoms over time or vice versa. Notably, depressive symptoms were significantly associated with OOF status (i.e., adolescents' reporting having at least one OOF) cross‐sectionally, and they marginally significantly predicted OOF status over time, which suggests the possibility that teens with higher depressive symptoms may be more likely to turn to OOFs than their lower‐symptom peers. However, given that these results were only significant for cross‐sectional associations and marginally significant in the longitudinal model, perhaps they highlight the importance of examining specific qualities of these friendships, such as intimate disclosure, rather than the sole presence or absence of these friendships.

We found that when teens engaged in higher levels of intimate disclosure within their OOFs, they also reported elevated levels of depressive symptoms longitudinally. However, depressive symptoms did not predict intimate disclosure, suggesting a possible pattern of temporal precedence such that teens who choose to self‐disclose personal and intimate information about themselves show increases in their depressive symptoms over time.

These findings reveal possible implications of OOFs for mental health, particularly when characterized by high levels of intimate disclosure. It is plausible that adolescents experiencing higher levels of depressive symptoms turn to social media for cultivating friendships in the digital era, some of which may indeed involve meaningful connections. It is also possible that the positive effects of online friendships on mental health may be contingent upon relationship factors we did not directly assess, such as trust. It is possible that some forms of intimate disclosure, such as co‐rumination or excessive self‐disclosure, may be detrimental (Battaglini et al., 2021). Co‐rumination, defined as excessively engaging in ruminative discussions of negative thoughts and feelings, has been documented in digital settings and linked to higher online self‐disclosure (Battaglini et al., 2024; Stevic et al., 2025). Indeed, support‐seeking online could be one mechanism by which teens engage in excessive amounts of intimate disclosure with OOFs; online support‐seeking has previously been linked to higher levels of depressive symptoms (Mackenzie et al., 2023). Importantly, prior studies examining the associations between online self‐disclosure and co‐rumination have not specifically examined these constructs in the context of OOFs, and our study's measure of intimate disclosure may fail to capture perceived social support or levels of co‐rumination in these friendships. Previous cross‐sectional work with young adults (aged 18–30) comparing face‐to‐face and social media‐based emotional support has found that while face‐to‐face support was associated with lower odds of depression, social media‐based support was linked to higher odds of depression (Shensa et al., 2020). Although this prior study focused broadly on social media interactions and did not specifically assess OOFs, its findings align partially with our longitudinal results, suggesting that OOFs characterized by higher emotional investment may lead to higher rates of depression over time.

There is limited research on friendships exclusive to digital platforms, but one study found that such friendships may be protective for suicidal teens specifically (Massing‐Schaffer et al., 2022). This suggests that OOFs could be particularly beneficial for individuals lacking in‐person social support and struggling with mental health challenges, which may be in line with our cross‐sectional findings. For nonclinical populations (e.g., the current study), these buffering effects may be attenuated. One large‐scale cross‐sectional survey in the UK differentiated between online‐only and in‐person friendships in teens aged 11–16, finding that communication with OOFs was negatively associated with well‐being, whereas online communication with friends known offline had a positive association—even after controlling for friendship quality (Anthony et al., 2023). While these findings do not directly address depressive symptoms or imply directionality, our longitudinal results suggest that OOFs may not be protective against depressive symptoms and, in cases of high intimate disclosure, could exacerbate them. One possibility is that OOFs are associated with a broader increase in online time and engagement, which may have complex implications for depressive symptoms. For instance, social displacement theory proposes that increased time spent on social media may be linked to decreases in in‐person interactions (Hall & Liu, 2022; Kushlev et al., 2019; Vacchiano & Valente, 2021). This may be especially true for teens who struggle with social connectedness in their in‐person friendships and who use digital spaces for social connection, which in turn may reduce time spent doing other activities associated with better well‐being, such as sleep or physical activity (Scott & Woods, 2019; Viner et al., 2019; Winstone et al., 2021).

## LIMITATIONS AND FUTURE DIRECTIONS

These results offer a preliminary glance at how OOFs might be a risk factor for some youth, especially when characterized by high levels of intimate disclosure. Nevertheless, future work should address some of the limitations of the current short‐term longitudinal study. First, to better understand the direction of effects between OOFs and the development of depression, further longitudinal investigations should employ multiple data points spanning over longer timeframes. Additionally, methods such as ecological momentary assessments could disentangle the nuances of digital friendship‐building and mental health symptoms in real time. Since our sample only had one follow‐up point and relied on self‐report questionnaire measures, we were unable to capture longer‐term prospective associations between our variables, and our measures may have been subject to recall bias. Similarly, although we controlled for baseline levels of OOF status, depressive symptoms, and intimate disclosure in our longitudinal regressions, we may have missed important nuances related to OOF stability, such as formation and dissolution of OOFs between time points. Thus, ecological or more frequent assessments of OOF status and intimate disclosure could better capture these temporal dynamics. It is also important to consider that the timing of data collection in the current study was just before the COVID‐19 pandemic, when OOFs may have been less prevalent or less essential as a source of social support for adolescents. It is possible that during school closures and social distancing measures, adolescents relied on digital settings to connect with others more heavily than before, increasing their reliance on OOFs and changing their impact on well‐being. Although our sample was large, school‐based, and ethnically diverse, with over half of participants identifying as Hispanic/Latinx, it still lacked broader racial diversity, which may limit the generalizability of our findings. Another important direction for future research will be to consider adolescents' offline friendships in the context of their OOFs; important developmental differences may emerge between youth who have offline support in addition to OOF support versus those who only have the support of online friends. Furthermore, while our assessment of intimate disclosure captured an important aspect of friendship quality using an adapted version of a well‐replicated and validated scale, incorporating more thorough assessments of stability, frequency, and duration of social connectedness within OOFs is also important. Future work should also assess more details regarding reciprocity in OOF intimate disclosure that may lead to co‐rumination, in addition to including broader measures of mental health symptoms. These findings may also shed light on existing literature suggesting that the adolescents who are most susceptible to developing depressive symptoms might concurrently experience heightened levels of both the advantages and disadvantages of social media engagement (Flannery et al., 2022). Additionally, future work can focus more on understanding the qualities of these digital friendships, which could inform interventions aimed at promoting adolescent well‐being in the digital age while maximizing benefits and minimizing risks for especially vulnerable youth.

## AUTHOR CONTRIBUTIONS

**Jennifer S. Silk:** Writing – review and editing. **Jacqueline Nesi:** Writing – review and editing; methodology. **Zelal Kilic:** Conceptualization; writing – original draft; writing – review and editing; formal analysis. **Sophia Choukas‐Bradley:** Supervision; investigation; project administration; methodology; writing – review and editing; conceptualization. **Anne J. Maheux:** Data curation; formal analysis; writing – review and editing; investigation; methodology.

## CONFLICT OF INTEREST STATEMENT

JN writes Techno Sapiens, a psychology newsletter for which she receives subscription and sponsor payments. She is co‐owner of Tech Without Stress, LLC, which provides free and paid resources for parents raising children in the digital age. The authors declare no other conflicts of interest.

## ETHICAL APPROVAL STATEMENT

All data collection procedures were deemed exempt by Advarra, the Character Lab Research Network's external IRB (PRO00028576; dated September 19, 2019). All secondary data analyses were deemed exempt by the University of Delaware IRB (protocol no.: 1630562–1; dated September 2, 2020). All authors complied with ethical standards for conducting research with human subjects.

## PATIENT CONSENT STATEMENT

Parental consent was not collected per Character Lab Research Network (CLRN) data collection procedures because: (1) CLRN had been designated a School Official under FERPA and formalized through written legal agreements; (2) access to students' parents could not be gained in a reasonable manner; (3) the study posed no more than minimal risk; and (4) all topics protected under the Protection of Pupil Rights Amendment were prohibited. CLRN provided an informational parent letter for schools to share with parents. Adolescent participants provided assent prior to participation. During the assent process, participants were informed that they could withdraw from the study at any time for any reason.

## Data Availability

The data that support the findings of this study are unavailable due to privacy or ethical restrictions.
